# Monetary incentives do not reduce the repetition-induced truth effect

**DOI:** 10.3758/s13423-021-02046-0

**Published:** 2021-12-16

**Authors:** Felix Speckmann, Christian Unkelbach

**Affiliations:** Social Cognition Center Cologne, University of Cologne, Richard-Strauss-Straße 2, 50931 Köln, Germany

**Keywords:** Truth effect, Repetition, Fake news, Cognitive illusions, Misinformation, Incentivized responding, Conspiracy theories

## Abstract

People rate and judge repeated information more true than novel information. This truth-by-repetition effect is of relevance for explaining belief in fake news, conspiracy theories, or misinformation effects. To ascertain whether increased motivation could reduce this effect, we tested the influence of monetary incentives on participants’ truth judgments. We used a standard truth paradigm, consisting of a presentation and judgment phase with factually true and false information, and incentivized every truth judgment. Monetary incentives may influence truth judgments in two ways. First, participants may rely more on relevant knowledge, leading to better discrimination between true and false statements. Second, participants may rely less on repetition, leading to a lower bias to respond “true.” We tested these predictions in a preregistered and high-powered experiment. However, incentives did not influence the percentage of “true” judgments or correct responses in general, despite participants’ longer response times in the incentivized conditions and evidence for knowledge about the statements. Our findings show that even monetary consequences do not protect against the truth-by-repetition effect, further substantiating its robustness and relevance and highlighting its potential hazardous effects when used in purposeful misinformation.

People see, read, and hear many different facts and statements each day (e.g., news, social media, conversations), which they can believe or doubt. Apparently, people use repetition as a cue to make this judgment; thus, believing repeated statements more compared with nonrepeated statements, a phenomenon known as the illusory truth effect, a truth-by-repetition effect, or simply a truth effect (Brashier & Marsh, 2020; Unkelbach et al., 2019).

In the seminal work by Hasher et al. (1977), the authors presented participants with 60 statements in three different sessions, 2 weeks apart each. Half of these statements were true (e.g., “Kentucky was the first state west of the Alleghenies to be settled by pioneers.”) and half of them were false (e.g., “Zachary Taylor was the first president to die in office.”). During each session, 20 of the statements were repeated (i.e., shown at every session) and the remaining 40 were new. After the presentation phase in each session, participants rated the validity of each statement. The authors found that participants judged repeated statements as more valid than new statements, demonstrating the basic truth effect.

Since then, a large body of research has replicated the original effect and investigated different explanations, mediators, and moderators (for a meta-analysis, see Dechêne et al., 2010; for recent summaries, Brashier & Marsh, 2020; Unkelbach et al., 2019). The effect has gained more prominence over the last years, as it may serve as an explanation for people’s belief in conspiracy theories, misinformation, and fake news, due to the frequent repetition of false information on the internet and social media (Pennycook et al., 2018; Vosoughi et al., 2018). In addition, repetition trumps even knowledge about a given state of affairs (Fazio et al., 2015). However, virtually all truth effect studies relied on self-reports of subjective truth, validity, or belief, without consequences for participants. Here, we investigate what happens if a given decision (i.e., “true” or “false”) has monetary consequences for the decision-maker. In other words, on a functional level, we ask if the truth effect persists if participants’ decisions are (highly) incentivized.

The reasoning behind this approach is straightforward. Without consequences, participants might have little motivation to provide correct assessments of their internal states (i.e., “Do you believe this statement?”) nor to invest too much effort into correct responses (i.e., “Is this true or false?”). In particular, when research employs online surveys, participants are likely not highly motivated to perform to the fullest of their ability. This “cognitive miser” perspective (Kurzban et al., 2013; Zipf, 1949) would predict that participants judge statements heuristically, relying on more superficial cues such as repetition and the resulting familiarity or processing fluency (see Unkelbach et al., 2019). However, if beliefs have consequences via “true/false” decisions in the form of incentives for these decisions, participants could invest more effort and potentially recall and consider more relevant knowledge that would lead to correct judgments.

Incentives as a way to increase effort are well established and can be derived from several classic theories, such as expectancy theory (Vroom, 1964), agency theory (Eisenhardt, 1989), or goal-setting theory (Locke et al., 1981). Depending on the task’s nature, the increased effort may also lead to increased performance (Bonner & Sprinkle, 2002). However, previous research has shown that the influence of incentives on several cognitive biases is small ( e.g., base rate neglect, anchoring; Enke et al., 2021; Speckmann & Unkelbach, 2021). Nevertheless, observed bias reductions were mainly due to reduced reliance on intuition and reduced reliance on superficial cues, which should also reduce the effect of repetition on judged truth. Furthermore, incentives increased response times, indicating increased effort. As we use a statement set for which participants have some knowledge (Unkelbach & Stahl, 2009), increased effort implies that participants try harder and try longer to remember relevant knowledge to judge the statements.

If incentives decrease the truth effect, it would suggest that the real-life impact of repeating information is less critical than assumed so far. People likely invest some mental effort into decisions with consequences, and if such effort reduces the truth effect, it would shift the research focus on beliefs and decisions that people consider only superficially. However, if monetary incentives do not reduce the truth effect, it would underline the relevance of the phenomenon for real-life scenarios and decisions with consequences. On the theoretical level, it would show that people potentially consider repetition and its processing consequences, such as familiarity or fluency, as valid cues for their decisions (see Unkelbach & Greifeneder, 2018).

## The present research

We used a standard truth effect research procedure (e.g., Bacon, 1979; Garcia-Marques et al., 2015; Unkelbach & Rom, 2017). Participants read statements in a presentation phase, half factually true and half factually false, and a judgment phase, where participants judged in a binary-forced choice format if a given statement is “true” or “false.” Going beyond previous research, we added monetary consequences to participants’ choices: Correct responses added points and incorrect responses deducted points; these points directly translated into a monetary bonus of up to 12 Euro in a high incentives condition, 6 Euro in a medium incentives condition, and no monetary bonus in a control condition.

Given the considerations above, monetary incentives may influence the truth effect in two ways. First, participants could try to retrieve more relevant information about the presented statements. In signal detection theory terms (Swets et al., 2000), this should increase participants’ discrimination ability between factually true and factually false statements. Second, participants could try to avoid extraneous influences on their judgments, such as repetition. In signal detection theory terms, this should decrease participants’ response bias for repeated compared with new statements.

We used the statement set by Unkelbach and Stahl (2009), who showed that participants have some knowledge regarding these statements and respond more frequently “true” to repeated statements. The experiment was preregistered, and we report how we determined our sample size, all data exclusions (if any), all manipulations, and all measures (the preregistration, data, and materials can be found at: https://osf.io/8sj4r/).

## Method

### Materials

We used 120 statements (60 true, 60 false) from Unkelbach and Stahl (2009). Table 1 shows some example statements.

**Table 1:** Examples of statements used in the experiment

Correct statements	Incorrect statements
The first windmills were built in Persia. The cat is the only pet that does not appear in the Bible. The painting *Bal du moulin de la Galette* was painted by Renoir. The name of the Russian space station MIR means “peace.” Alberto Fujimori was the Japanese president from 1990 to 2000.	Henbane was a popular spice during the Middle Ages. The world’s most expensive colorant is true ultramarine. Volcanos can have a theoretical maximum elevation of about 5,000 meters. Adelaide is Australia’s oldest city. The world’s largest lake is the Aral Sea.

### Participants and design

We had no a priori estimate for the effect size of monetary incentives; we pragmatically aimed for 100 participants per condition as an established threshold in our lab (i.e., smaller effects are too costly to investigate). In the end, we recruited 321 participants on campus (*M*_age_ = 23.09 years, *SD* = 6.84; 180 female, 141 male) who participated in exchange for 4€ plus a potential bonus in the incentivized conditions. In the two incentivized conditions, participants could earn up to 12€ (high incentives condition) or 6€ (medium incentives condition), but we recruited all participants with the expectation of receiving 4€. They were randomly assigned to the high incentives, medium incentives, and no incentives conditions. There were 110 participants in the high incentive, 105 participants in the medium incentive, and 106 participants in the no incentive conditions. Half of the statements were randomly sampled per participant to be shown in the presentation phase (i.e., “old” statements compared with “new” statements in the judgment phase). Half of the statements were factually true and half factually false; the other half only appeared in the judgment phase. Thus, participants judged 30 true-old, 30 false-old, 30 true-new, and 30 false-new statements.

### Procedure

Experimenters approached participants on campus, led them to the laboratory, and seated them in front of a computer with a Visual Basic program already running. The program asked participants to enter their age, gender, and to indicate whether German is their native language, first foreign language, or second foreign language. The program then asked participants to turn off their cell phones to avoid cheating and explained the general setup of the experiment. Specifically, it told participants, “In the first part, you will see a list of statements. Please try to read all of the statements, even if the presentation is quick. By doing this, we want to examine certain memory processes. After that, we will continue with the judgment of statements. For each statement, please indicate by keypress whether the statement is TRUE or FALSE.” In the high and medium incentives conditions, this explanation continued, “ATTENTION: During the judgment phase, you can earn up to 12€ (6€) extra. This will be explained later.”

After that, the presentation phase started. To begin, participants pressed the space key, and the program stated before the presentation phase: “Please note that one-half of the statements are true and the other half is false.” The program randomized statements anew for each participant; each statement appeared on-screen for 1.5 seconds with a pause between statements of 1 s. After the presentation phase, the program continued with further explanations: “We will now continue with the judgment of the statements. To this end, you will be repeatedly presented with a statement and have to decide if it is true or false. Two keys of the keyboard are marked. You can decide by using these keys. YES–TRUE: left key, NO–FALSE: right key. The key mapping will also be visible on screen.” The following part dealt with the bonus payments and was only displayed to participants in the high and medium incentives conditions: “By answering correctly or incorrectly, you can win or lose real money that will be added to your point balance. For a correct TRUE/FALSE answer, you will receive 10 (5) cents. For an incorrect answer, you will lose 10 (5) cents. You will judge 120 statements and can thus earn up to 12 (6)€! Your point balance cannot turn negative. At the end of the study, you will receive your basic compensation of 4€ on top of your point balance and see a summary of all of your answers.”

After this explanation, the judgment phase began. The program asked participants to place their fingers on the marked keys (“y” and “-” on a German keyboard) and to start by pressing the space key. The judgment phase presented 120 statements, and each statement was displayed until participants pressed either one of the response keys. After the judgment phase, the program debriefed participants and showed them a summary of all questions, whether their response was correct, and how many cents (if any) they received for each question. Participants then showed the ending screen to the experimenter, who thanked participants and paid them according to their performance in the medium and high incentives conditions.

## Results

### Percentage of “true” judgments (PTJs)

**Fig. 1:**
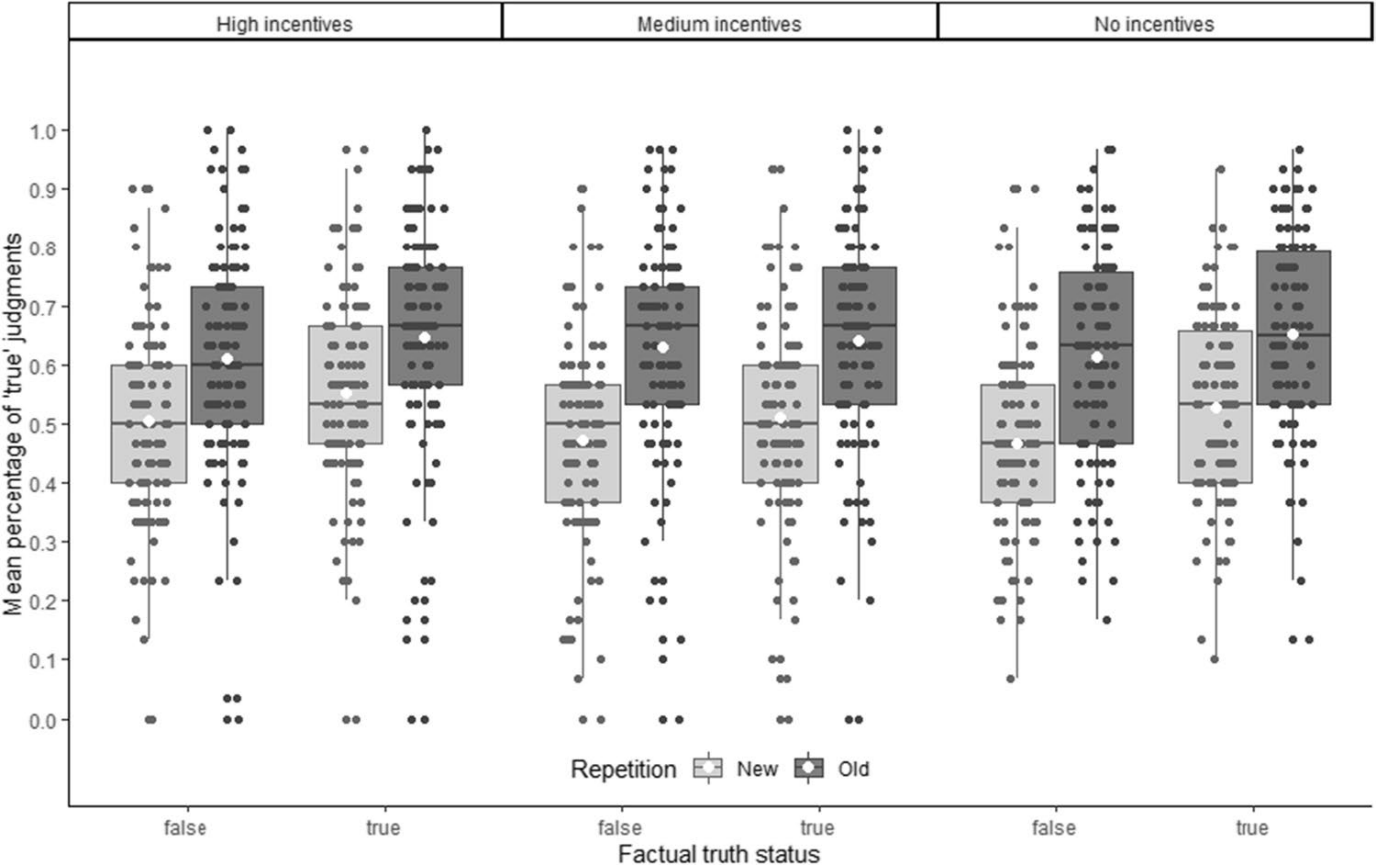
Mean percentage of “true” judgments as a function of repetition (old vs. new) and factual truth status (true vs. false), separated by incentives (High vs. Medium vs. None). The white dots represent the means, the black horizontal lines represent the medians, the boxes represent the 25% quartiles, the whiskers extend to the highest (lowest) point within the interquartile range (i.e., the distance between first and third quartile)

To analyze the influence of incentives on the truth effect, we computed the percentage of “true” judgments (PTJs) for each participant by coding “true” judgments as 1 and “false” judgments as 0, and averaging the responses across the 120 decisions, separately for the four combinations of factual truth (i.e., true and false) and repetition (i.e., new vs. old). Higher PTJ values indicate a higher likelihood of responding “true” for a given statement (see Unkelbach & Rom, 2017). We then submitted the PTJs to a 2 (repetition: old vs. new) × 2 (factual truth status: true vs. false) × 3 (incentive: high vs. medium vs. none) mixed analysis of variance (ANOVA) with repeated measures on the first two factors. Figure 1 shows the respective means.

As Fig. 1 suggests, this analysis replicated the standard truth effect. Participants showed higher PTJs for old statements (*M* = 0.633, *SD* = 0.182) compared with new statements (*M* = 0.507, *SD* = 0.168), *F*(1, 318) = 182.31, *p* < .001, *η*_p_^2^ = .364. Participants also showed knowledge about the statements with higher PTJs for factually true statements (*M* = 0.589, *SD* = 0.180) compared with factually false statements (*M* = 0.550, *SD* = 0.190), *F*(1, 318) = 65.00, *p* < .001, *η*_p_^2^ = .170. However, there was no significant main effect for incentives, *M*_*high*_ = 0.579, *SD*_*high*_ = 0.148, *M*_*med*_ = 0.564, *SD*_*med*_ = 0.145, *M*_*no*_ = 0.565, *SD*_*no*_ = 0.127, *F*(2, 318) = 0.40, *p* = .669, *η*_p_^2^ = .003, and neither the knowledge effect nor the repetition-induced truth effect interacted with the incentives condition, *F*(2, 318) = 2.25, *p* = .107, *η*_p_^2^ = .014, and *F*(2, 318) = 2.07, *p* = .128, *η*_p_^2^ = .013, respectively.

In addition, the preregistered polynomial contrasts (linear and quadratic) did not interact with the repetition effect or the knowledge effect, *t*(318) = 1.49, *p* = .136, *d* = 0.17, and *t*(318) = 0.21, *p* = .837, *d* = 0.02, for the linear trends, and *t*(318) = −1.37, *p* = .173, *d* = −0.15, and *t*(318) = 1.84, *p* = .066, *d* = 0.21, for the quadratic trends.

To further explore the influence of incentives on PTJs, we also used an additional contrast testing the incentive conditions against the no incentives condition, coded -2, +1, +1, for the no, medium, and high incentive conditions, respectively. For the knowledge effect, this contrast showed no influence, *F*s < 1. For the truth effect, however, the second contrast showed a significant effect, *t*(1, 318) = 1.99, *p* = .048, *d* = 0.22, indicating a slightly smaller truth effect in the incentive conditions (*M* = 0.121, SD = 0.157) compared with the no incentive condition (*M* = 0.134, *SD* = 0.187). However, this test should be treated with caution due to the exploratory nature of this contrast and the small effect size. Furthermore, after excluding two participants who responded “false” to all questions (flat responding), the *p* value of this contrast changes to *p* = .050 and thus became nonsignificant by conventional standards.^1^

The only other significant effect was an interaction of factual truth and repetition, *F*(1, 318) = 4.75, *p* = .030, *η*_p_^2^ = .015. The truth effect was stronger for factually false statements (*M* = 0.136, *SD* = 0.190) compared with factually true statements (*M* = 0.115, *SD* = 0.184). This effect conceptually replicates the pattern by that repetition has stronger effects on false, and thereby necessarily unknown, information (see Hasher et al., 1977; Unkelbach & Speckmann, 2021).

Because the lack of significant incentive effects on truth judgments does not provide evidence for the absence of the effect, and because the *p* value for the −2, 1, 1 contrast was close to the alpha level, we complemented both analyses with Bayesian analyses. As we did not preregister any priors, we used default priors for both analyses. For the main effect of incentives, we used JASP in Version 0.15 (JASP Team, 2021). A Bayesian repeated-measures ANOVA showed a Bayes factor of BF_01_ = 20.632, commonly seen as strong evidence for the H_0_ (Jarosz & Wiley, 2014). As JASP lacks functionality for custom contrasts, we used R (R Core Team, 2020) with the packages rstanarm (Goodrich et al., 2020) and bayestestR (Makowski et al., 2019) for the exploratory −2, 1, 1 contrast. This analysis showed a Bayes factor of BF_01_ = 6.410, commonly seen as positive or substantial evidence for the H_0_ (Jarosz & Wiley, 2014). The JASP file containing the analysis and the relevant R code are accessible from the OSF project linked above.

### Correctness

To summarize the incentive influence on decisions, we also analyzed the effect of incentives on the overall correctness of the judgments (i.e., “true” judgment of a factually true statement or “false” judgment of a factually false statement), which provides a direct estimate of the incentive effect on decision correctness. To this end, we computed a variable indicating the correctness of each decision and submitted the average correctness, varying from 0 (*never correct*) to 1 (*always correct*) to a one-way ANOVA with incentives (high vs. medium vs. none) as the between factor. The main effect for incentives was not significant, *F*(2, 318) = 2.25, *p* = .107, *η*_p_^2^ = .014, and neither was the linear trend, *t*(318) = 0.69, *p* = .489, *d* = 0.08. However, the quadratic trend was significant, *t*(318) = 2.01, *p* = .045, *d* = 0.23. Participants were less frequently correct in the medium incentive condition (*M* = 0.513, *SD* = 0.041), compared with the no incentives (*M* = 0.525, *SD* = 0.043) and the high incentive condition (*M* = 0.521, *SD* = 0.046). Due to the small effect size and the nonpredicted pattern, we hesitate to interpret this effect.

### Latencies

**Fig. 2:**
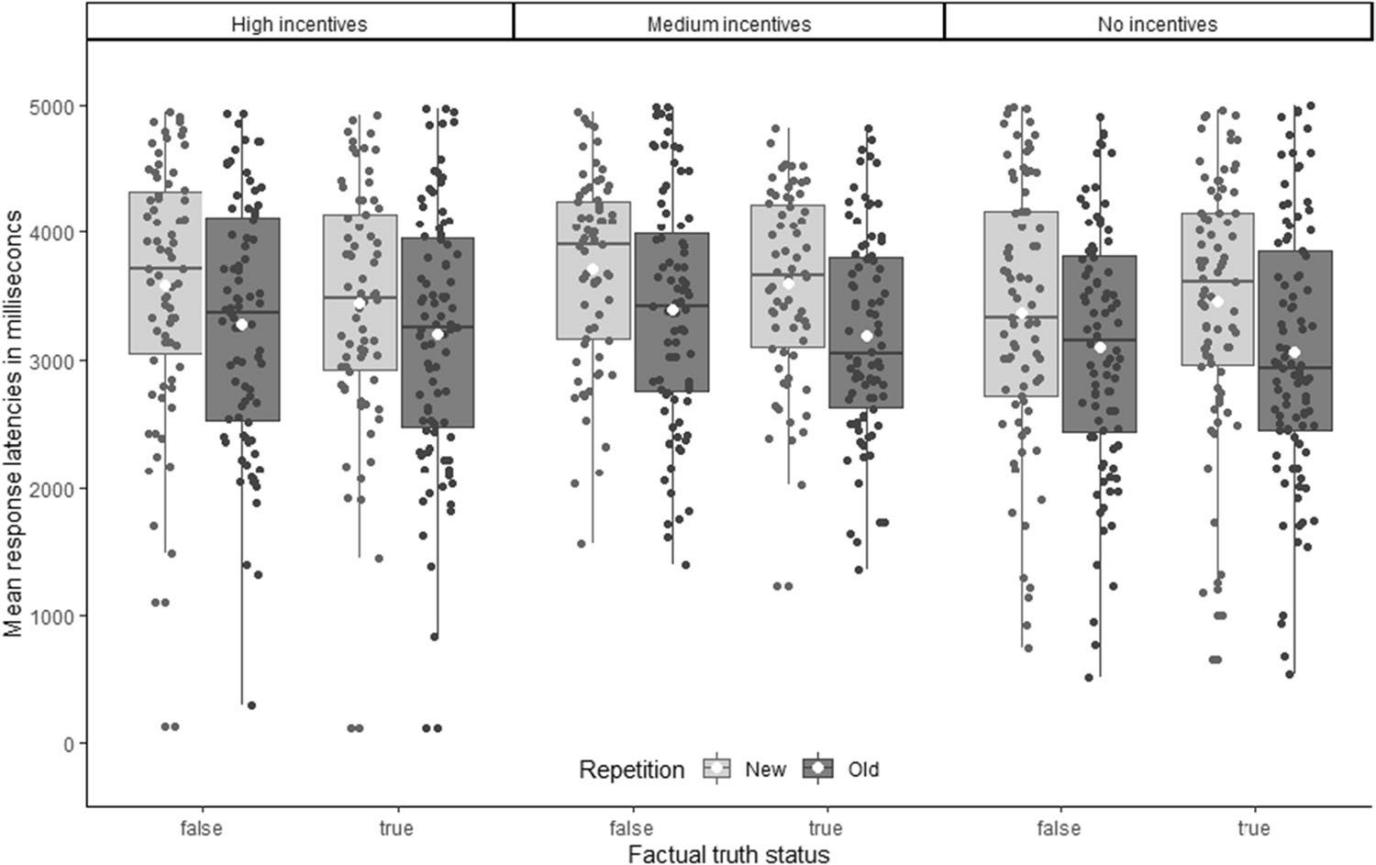
Mean response latencies as a function of repetition (old vs. new) and factual truth status (true vs. false), separated by incentives (High vs. Medium vs. None). Error bars represent standard errors of the means. The white dots represent the means, the black horizontal lines represent the medians, the boxes represent the 25% quartiles, the whiskers extend to the highest (lowest) point within the interquartile range (distance between first and third quartile)

We analyzed participants’ raw (i.e., no trimming or transformation) response latencies in millisecond the same way as the PTJs. Figure 2 shows the respective means. As Fig. 2 indicates, participants responded faster to old statements (*M* = 3794, *SD* = 1572) compared with new statements (*M* = 4433, *SD* = 1691), *F*(1, 318) = 188.36, *p* < .001, *η*_p_^2^ = .372. In addition, participants responded faster to factually true statements (*M* = 4022, *SD* = 1556) compared with factually false statements (*M* = 4205, *SD* = 1759), *F*(1, 318) = 22.32, *p* < .001, η_p_^2^ = .066, underlining that participants possessed some knowledge. In addition, incentives significantly influenced the overall latencies (*M*_*high*_ = 4236, *SD*_high_ = 1595; *M*_*med*_ = 4312, *SD*_med_ = 1490; *M*_*no*_ = 3790, *SD*_no_ = 1375), *F*(2, 318) = 3.80, *p* = .023, η_p_^2^ = .023.

To explore the influence of incentives on latencies, we used the same two contrasts for the PTJs, one testing a linear influence of incentives and one testing the two incentive conditions against the no incentives condition. Only the linear contrast showed a significant effect, *t*(318) = 2.20, *p* = .029, *d* = 0.25, indicating that participants took on average more time for their true–false decisions in the high incentives condition (*M* = 4236, *SD* = 1738) compared with the no incentives condition (*M* = 3789, *SD* = 1484). Again, these contrasts are post hoc and should not be treated as confirmatory evidence.

### SDT analyses

As preregistered, we also analyzed the response rates with a signal-detection theory (SDT) analysis (see Unkelbach, 2006, 2007). The SDT analysis is particularly suited for the present task, as it delivers two parameters, *d'* and β, which are directly interpretable as knowledge and the truth effect, respectively, in the present design. An interaction with incentives may indicate an influence of incentive on participants’ higher reliance on knowledge or avoidance of bias (i.e., the truth effect). However, *d'* and β did not significantly differ as a function of incentives, replicating the PTJ analyses. For the complete analysis, please refer to the supplemental materials on OSF (https://osf.io/x8wf2/).

## Discussion

The present study investigated the influence of true–false judgments’ monetary consequences in a repetition-induced truth paradigm. We speculated that the monetary consequences might increase discriminability or reduce bias, thereby reducing the influence of repetition on judged truth. We replicated a typical truth effect and also the knowledge effect by Unkelbach and Stahl (2009). However, although participants could receive a bonus of up to 12€ in the high incentives condition and 6€ in the medium incentives condition, these monetary incentives did not substantially influence the truth effect or the knowledge effect. Using an exploratory contrast, we found a slight difference in the truth effect between the two incentive conditions and the no-incentive condition: Participants showed a slight reduction in their tendency to judge repeated information as true. Given the small effect size and the fact that this contrast was not preregistered, it should not be interpreted as strong evidence. If there is an effect of incentives on responses in the truth effect paradigm, it is likely minimal.

Despite the overall nonsignificant influences of incentives on the truth effect, it seems that our manipulation had the intended effect. The significant differences in response times between different incentive levels suggest that participants were more motivated to respond correctly as much as possible and consequently spent more time judging the statements.

However, it is worth noting that timing may play a part in the present patterns. Research by Jalbert et al. (2020) showed that warning participants that half of the statements are false reduces the truth effect in a truth-by-repetition paradigm. However, the warning is only effective if shown prior to exposure and ineffective when shown only before the judgment phase (see also Brashier et al., 2020). Similarly, Lewandowsky et al. (2012) recommend inserting warnings prior to the presentation phase to help people resist misinformation.

In our experiment, participants knew that their performance would be incentivized and the maximum amount of money they could earn (i.e., in the incentives conditions), but they did not concretely know how responding would be incentivized (i.e., how much money could be gained or lost for each question). This unclarity suggests the possibility that incentives might be more effective if explained in more detail prior to the presentation phase. However, before the presentation phase, participants knew that half of the statements would be false, as in the effective warning condition by Jalbert et al. (2020). Nevertheless, within the present setup, incentives neither increased participants’ retrieval of relevant material from memory nor decreased their reliance on repetition as a cue for truth.

These results thus further illustrate the robustness of the truth effect by showing that even adding direct consequences to people’s truth judgments does not affect it. These results are relevant as one may argue that the truth effect is often investigated with online samples of participants who might not care about their judgments because high or low performance is inconsequential. However, our data shows that the truth effect persists even when incentivizing laboratory participants with considerable amounts of money, ruling out this explanation. Our results also dovetail with a preprint manuscript by Brashier and Rand (2021); they also used a truth-by-repetition paradigm and found no effect of incentivizing a single random trial within 16 truth judgments on the truth effect, despite the fact that they provided repeated reminders about the potential reward.

Our data also fits well with existing research on other cognitive illusions, showing that incentives increase effort but not performance (Enke et al., 2021) and cognitive explanations of the truth effect. For example, the processing fluency explanation suggests that repeatedly seeing a piece of information makes it easier to process. This experienced processing fluency then serves as a cue to judge a piece of information as more true (Begg et al., 1992; Reber & Schwarz, 1999). While fluency may often be an ecologically valid cue for trueness (Reber & Unkelbach, 2010), people can also learn to use fluency as a cue for falseness (Corneille et al., 2020; Unkelbach, 2007). However, participants in the present experiment had no reason to doubt the ecological validity of fluency as a cue for truth, and thus effort did not decrease their reliance on fluency. In terms of an incentives–effort–performance link (Bonner & Sprinkle, 2002), we provide evidence for the incentives–effort link, but the effort–performance link is disrupted, possibly due to the truth effect’s nature.

Thus, our data support existing cognitive explanations of the truth effect with potential implications for real-world phenomena (e.g., fake news, conspiracy theories, strategic misinformation): Even people who should be motivated to judge truth correctly still fall prey to the truth effect.

## Data Availability

The data sets generated during and/or analyzed during the current study and all materials used are available on the OSF (https://osf.io/8sj4r/).

## Footnotes

1 We repeated all analyses with the two exclusions, but none of the patterns (PTJs, correctness, or latencies) changed apart from the explorative contrast for PTJs. As we did not preregister this exclusion, we report the rest of the data without exclusions.
